# Replication of GWAS Coding SNPs Implicates MMEL1 as a Potential Susceptibility Locus among Saudi Arabian Celiac Disease Patients

**DOI:** 10.1155/2015/351673

**Published:** 2015-12-30

**Authors:** Omar I. Saadah, Noor Ahmad Shaik, Babajan Banaganapalli, Mohammed A. Salama, Sameer E. Al-Harthi, Jun Wang, Harbi A. Shawoosh, Sharifa A. Alghamdi, Yagoub Y. Bin-Taleb, Bakr H. Alhussaini, Ramu Elango, Jumana Y. Al-Aama

**Affiliations:** ^1^Department of Pediatrics, Faculty of Medicine, King Abdulaziz University, Jeddah 21589, Saudi Arabia; ^2^Princess Al-Jawhara Al-Brahim Center of Excellence in Research of Hereditary Disorders, King Abdulaziz University, Jeddah, Saudi Arabia; ^3^Department of Genetic Medicine, Faculty of Medicine, King Abdulaziz University, Jeddah, Saudi Arabia; ^4^Department of Biology, Faculty of Science, King Abdulaziz University, Jeddah, Saudi Arabia; ^5^Department of Pharmacology, Faculty of Medicine, King Abdulaziz University, Jeddah, Saudi Arabia; ^6^BGI, Beishan Industrial Zone, Yantian District, Shenzhen 518083, China; ^7^Department of Pediatrics, King Fahd Armed Forces Hospital, Jeddah, Saudi Arabia; ^8^Department of Pediatrics, Division of Gastroenterology, King Faisal Specialist Hospital and Research Center, Jeddah, Saudi Arabia

## Abstract

Celiac disease (CD), a gluten intolerance disorder, was implicated to have 57 genetic susceptibility loci for Europeans but not for culturally and geographically distinct ethnic populations like Saudi Arabian CD patients. Therefore, we genotyped Saudi CD patients and healthy controls for three polymorphisms, that is, Phe196Ser in IRAK1, Trp262Arg in SH2B3, and Met518Thr in MMEL1 genes. Single locus analysis identified that carriers of the 518 Thr/Thr (MMEL1) genotype conferred a 1.6-fold increased disease risk compared to the noncarriers (OR = 2.6; 95% CI: 1.22–5.54; *P* < 0.01). This significance persisted even under allelic (OR = 1.55; 95% CI: 1.05–2.28; *P* = 0.02) and additive (OR = 0.35; 95% CI: 0.17–0.71; *P* = 0.03) genetic models. However, frequencies for Trp262Arg (SH2B3) and Phe196Ser (IRAK1) polymorphisms were not significantly different between patients and controls. The overall best MDR model included Met518Thr and Trp262Arg polymorphisms, with a maximal testing accuracy of 64.1% and a maximal cross-validation consistency of 10 out of 10 (*P* = 0.0156). Allelic distribution of the 518 Thr/Thr polymorphism in MMEL1 primarily suggests its independent and synergistic contribution towards CD susceptibility among Saudi patients. Lack of significant association of IRAK and SH2B3 gene polymorphisms in Saudi patients but their association in European groups suggests the genetic heterogeneity of CD.

## 1. Introduction

Celiac disease (CD) is a T-cell-mediated inflammatory disorder with underlying involvement of autoimmune, genetic, and environmental components [1, 2]. This chronic enteropathy is caused by permanent intolerance to ingested gluten from wheat, barley, and rye in genetically susceptible children and adults [3–5]. The common clinical manifestations presented by patients with CD are chronic diarrhea, abdominal distension, failure to thrive, and short stature [4, 6]. An approximate 1% prevalence rate of CD among Caucasians places them as the most vulnerable ethnic group for developing CD. However, current day improved knowledge about celiac disease prevalence and clinical and diagnostic characteristics has indicated that it is common not only in Europe, but also in developing countries where wheat is included as a major dietary component [4, 7]. In the Middle East, recent trends indicate the variable prevalence of CD from 0.14% to 1.17% in low risk and from 2.4% to 44% in high risk populations of Arab origin [8–10].

Celiac disease is a complex pathology where disease onset is governed by various genetic factors. The genetic liability to CD is evidenced by the higher rate of familial incidence and also by its strict linkage with some human leukocyte antigens (HLA) class 2 alleles [11]. Polymorphic HLA-DQ2 and/or HLA-DQ8 alleles have been identified as a prerequisite but they alone cannot justify the development of CD. Therefore, it is expected that non-HLA genetic loci play a role in celiac disease susceptibility. The introduction of genome-wide association studies (GWAS) and subsequent immunochip fine mapping studies has been a great breakthrough in bringing down the celiac disease risk loci (both HLA and non-HLA) to 57 SNPs. Together all these risk loci can only explain up to 54% of celiac disease risk leaving a subset of the genetic risk factors for celiac disease unexplained among certain case groups [12–18].

A common complication faced by geneticists in deriving GWAS based genotype-phenotype associations for complex traits like CD is that most of these studies were conducted in European patients, so they miss population-specific genetic effects in culturally and ethnically different disease groups. In addition to this GWAS sample sets consist of nonuniform control cohorts with differential contribution to disease association signal. The prediction of clinical relevance for disease associated GWAS loci is further complicated by the fact that the majority of SNPs are located in intronic or regulatory regions and they have no readily annotated gene function and therefore cannot be assigned to a particular biological pathway. On the contrary, SNPs occurring in coding regions are often able to alter amino acid sequence of polypeptides which in turn results in structural and functional changes in the protein encoded, hence the cellular functions. An important finding revealed by Trynka et al. [16] is that, on functional annotation of 57 genetic loci associated with celiac disease, only 3 genetic variations, that is, rs1059702 in IRAK1, rs3184504 in SH2B3, and rs3748816 in MMEL1, are found to occur in exonic regions and subsequently affect the amino acid sequences of concerned proteins.

The clinical ascertainment of real disease causal genetic variations from statistically correlated CD-GWAS loci requires data from population-specific replication studies. It is well agreed that conducting genetic studies in the mostly consanguineous Arabian population has a great potential to identify novel genetic connections and may also narrow down the number of real disease associated genes in complex diseases. To the best of our information, until today no study has examined the contribution of genetic variations in celiac disease among patients of Saudi Arabian origin. Therefore, the current study has aimed to replicate the association of three amino acid substitution SNPs identified by fine mapped loci of GWAS results of European populations to question their implication in CD development among Saudi Arabian patients.

## 2. Methods

### 2.1. Subjects and Sampling

Ethics approval for the present study was obtained from the Research Ethics Committee, King Abdulaziz University Hospital, Jeddah. Study participants comprised Saudi nationals of whom 97 were unrelated patients with celiac disease and 124 were self-reported healthy controls, who never reported any health complications including allergies. Diagnosis of celiac disease was made by testing for antibodies against gliadin and endomysium (EMA) or tissue transglutaminase (TGA) as well as a confirmatory small bowel biopsy as per the criteria set out by the European Society for Paediatric Gastroenterology, Hepatology and Nutrition (ESPHGAN). 2 mL of blood samples was collected for genetic analysis from each participant after obtaining their consent or approval.

### 2.2. Genetic Analysis

The DNA was isolated from whole blood samples by using a DNA blood mini kit (QIAamp, Qiagen, Inc., Valencia, CA, USA) following the manufacturer's instructions. Three SNPs, that is, rs1059702, rs3184504, and rs3748816, were genotyped using commercial TaqMan allelic discrimination assays (Applied Biosystems, Foster City, California, USA) on an ABI7500HT sequence detection system (Applied Biosystems). The TaqMan probe labeling is as follows: VIC dye (green fluorophore) was made correspondent to wild type allele, whereas FAM dye (blue fluorophore) was made for mutant allele. The emission of green color fluorescence during amplification cycle indicates homoallelic native type, blue color indicates homoallelic mutant type, and combination of both blue and green color fluorescence indicates the heteroallelic state. The scatter plot was prepared based on the automatically computed threshold cycle values of each sample corresponding to the signal of either FAM or VIC. The 10 *μ*L reaction mixture consisting of 5 *μ*L of TaqMan Genotyping Master Mix (which contains AmpliTaq Gold DNA Polymerase UP (Ultra Pure), dNTPs without dUTP, Passive Reference ROX dye, and optimized mix components), 0.25 *μ*L TaqMan SNP Genotyping Assay (which includes 300 nM of each specific oligonucleotide primer and MGB probe 200 nM), and 1 *μ*L of DNA (50 ng/*μ*L) was carried out in a Micro Amp optical 96-well reaction plate. The conditions for PCR included 60°C for 1 min and 95°C for 10 min followed by 40 cycles of 95°C for 15 s and 60°C for 1 min. Genotyping calling was done using SDS version 2.3 software.

### 2.3. Statistical Analysis

Statistical analyses were performed with GraphPad QuickCalcs, version 6.0 (GraphPad Software Inc., USA), online statistical software. For each SNP, allele and genotype frequencies were computed in celiac patients and controls. Statistically significant difference in allele and genotypes was determined using Pearson's standard chi-squared test, odds ratio (OR), and 95% confidence interval (CI). Hardy-Weinberg equilibrium was tested on a contingency table of observed versus predicted genotype distributions using a chi-squared test with one degree of freedom. The epistasis between three investigated polymorphisms and incidence of CD was examined by nonparametric and genetic model-free multifactor dimensionality reduction (MDR) data mining approach using open-source multifactor dimensionality reduction (MDR) software (MDR version 2.0 beta 5, http://www.epistasis.org/). Statistical significance was tested through 1,000 permutation tests (MDR permutation testing module 0.4.9 alpha) to compare observed testing accuracies with those expected under the null hypothesis of null association. A *P* value of 0.05 was considered statistically significant.

## 3. Results

Our study group comprised 57 adolescent and 40 childhood celiac disease patients. Gender distribution of disease group showed that females (54%) were slightly higher in number compared to their male counterparts. The mean age of disease onset among adults was 16 ± 4.9 years and in children it was 9.6 ± 4.9 years. The control group consisted of 140 adults, whose mean age was 29 ± 3 years. The common clinical complaints seen in celiac patients were as follows: chronic diarrhea (82%), anorexia (80%), abdominal distension (75%), and abdominal pain (31%). Among the autoimmune disorders that were presented in our CD group were type 1 diabetes mellitus (32%), autoimmune thyroiditis (8%), and systemic lupus erythematosus (3%). The other nonautoimmune mediated disorders seen in patient group were osteomalacia (5%), seizure disorders (4%), and Down syndrome (4%).

Regarding genotyping results, all the three tested SNPs (rs1059702, rs3184504, and rs3748816) were compatible to Hardy-Weinberg equilibrium in both CD patients and controls. The frequencies of the AA (Phe/Phe), AG (Phe/Ser), and GG (Ser/Ser) genotypes for IRAK1 rs1059702 SNP among celiac cases in this study were 23.71%, 24.74%, and 51.54%, respectively (Table 1).

The minor allele frequency of A was found to be 36.08. However, the celiac patients and healthy controls did not significantly differ in terms of their genotype distribution (*P* = 0.72) and allele distributions (*P* = 0.28) (Table 2). For SH2B3 SNP, frequencies of TT (Trp/Trp), TC (Trp/Arg), and CC (Arg/Arg) genotypes and T (Trp) and C (Arg) alleles were found to be insignificantly different between both the study groups when tested for recessive and dominant models of disease risk (*P* ≥ 0.51). The genotypes encoding Met/Met (TT), Thr/Met (TC), and Thr/Thr (CC) in MMEL1 rs3748816 SNP have indicated that Thr/Thr genotype was significantly different between celiac cases (25.77%) and controls (12.09%) [*P* = 0.01; OR (95% CI) = 2.60 (1.22–5.54)]. In correspondence to this finding, the dominant model analysis has also revealed a significant association of Thr/Thr genotypes over Met/Met +Thr/Met genotypes with celiac disease risk [*P* = 0.003, OR (95% CI) = 0.35 (0.17–0.71)]. The minor allele C coding for Thr was significantly higher among celiac cases compared to controls [*P* = 0.02, OR (95% CI) =1.55 (1.05–2.28)].

To further elaborate our genotype findings, MDR analysis was applied to characterize high order gene-gene interactions in cases and controls.  Table 3 summarizes the results of exhaustive MDR analysis for combinations of the three polymorphisms studied for the risk of developing celiac disease. The best models will be accompanied by their testing accuracy, cross-validation consistency, and significant level determined by permutation testing. MDR results have revealed the interactions between MMEL1 (c.1553 T > C; p.Met518Thr) and SH2B3 (c.784 T > C; p.Trp262Arg) polymorphisms as the overall best model included with maximal testing accuracy of 63.18% and a maximal cross-validation consistency of 10 out of 10. This model was significant at the 0.0156 level. These results have reinforced the significant results of our single locus analysis, that is, MMEL1 as a risk factor for CD development in the Saudi Arabian population.

## 4. Discussion

The recent GWAS have implicated numerous genetic loci in celiac disease predisposition [12, 13, 17, 19]. However, genetic variations (in terms of allele frequency and linkage disequilibrium) and environmental factors (in terms of dietary patterns, alcohol, and smoking) often vary across different ethnicities and regions resulting in lack of consensus regarding the association of celiac disease susceptibility SNPs. Therefore, in the present work, we investigated the association of three nonsynonymous polymorphisms in MMEL1, IRAK1, and SH2B3 genes to enhance the likelihood of identifying alleles that are assumed to have functionally deleterious consequences with subsequent celiac disease risk predisposition in the Saudi Arabian population. The principle finding was that rs3748816 in MMEL1 is a significant contributor to celiac disease susceptibility in patients of Saudi Arabian ancestry. Although allele and genotype frequencies of IRAK1 and SH2B3 were insignificantly different between patients and controls, a potential synergistic effect was seen between MMEL1 and SH2B3 genes, reinforcing the significance of MMEL1 in celiac disease susceptibility. The compliance of three examined genetic polymorphisms with Hardy-Weinberg equilibrium in both patients and controls suggests that our results are unlikely to be biased by genotyping errors or population stratification.

The human MMEL1 gene encodes a type II class transmembrane protein (779 amino acids) belonging to metalloendopeptidases family, which is known to show ubiquitous expression. This gene is located on chromosome 1 and its size spans up to 0.4 MB with 24 exons. The genetic polymorphism rs3748816 is located in exon 16 of MMEL1 with the “T” allele codes (A**T**G) for the nonpolar hydrophobic amino acid methionine and the risk “C” allele codes (A**C**G) polar hydrophilic threonine at amino acid position 518. This change is predicted to be “probably damaging” based on the computational program Polymorphism Phenotyping (PolyPhen) [20]. This amino acid variant is located in alpha helix region close to peptidase family M13 N-terminal domain of MMEL1 protein. The MMEL1 protein in conjunction with other members of the metalloendopeptidase family appears to be involved in neuropeptide degradation [21]. The recent GWAS and follow-up case-control studies have identified the association of MMEL1 gene with rheumatoid arthritis (RA) susceptibility [22–24].

Growing evidence suggests that autoimmune diseases such as celiac disease and rheumatoid arthritis share at least 10 common genetic susceptibility loci [25]. Of those overlapping loci, rs4648562 (*r* ^2^ ≥ 0.89) of MMEL1 gene leads to splice site alteration of the concerned transcript. Although celiac disease and rheumatoid arthritis are distinct diseases by nature, they share common features such as association with HLA status, T-cell infiltration in target organs, and development of autoimmune antibodies. It is not clear what function this gene plays in gluten sensitivity if it is indeed a true disease associated gene. TNFRSF14 (rs2234167), a strong candidate for celiac diseases, which is less than 60 kb downstream is in strong linkage (*r* ^2^ = 0.89) with rs3890745 of MMEL1 further raising the possibility of its indirect involvement in celiac disease [26]. However, further work involving reverse genetics is required to fully establish the function of this gene and to ascertain how the disease-causative polymorphism(s) in MMEL1 gene exert their effect in celiac disease biology.

Although both IRAK1 (rs1059702) and SH2B3 (rs3184504) nonsynonymous genetic polymorphisms are statistically not associated with CD, this does not mean that they are not true disease susceptibility loci among Saudi Arabian patients. Independently these two loci are seen under strong linkage [by LD scores; *r* ^2^ > 0.9] with genetically critical regions for celiac disease development in Caucasians [14]. On either side (at least 0.2 MB) of the rs3184504 at 12q24 (Chr 12: 111833788 to Chr 12: 111932800) exist candidate genes like RNA processing factor ATXN2 and the nuclear localization inhibitor BRAP, which are known for their role in subclinical inflammation and lymphocyte activity regulation [27], and they were previously associated with CD susceptibility [13]. Indeed, rs3184504 was seen to be associated with expression status of SH2B3 in intestinal mucosa of patients with CD [28]. It is interesting to note that this polymorphism involves an amino acid substitution R262W in functionally important domain of protein that is known to be under positive natural selection and critical for efficient response to exogenous antigens [29]. The lack of significant association of rs3184504 in Saudi patients but its association in European groups [13, 16, 19] suggests the genetic heterogeneity of celiac disease.

We acknowledge some limitations in interpreting the present study results. Firstly, we have only screened 3 nonsynonymous genetic polymorphisms and did not cover the 50+ remaining SNPs (noncoding SNPs) reported by dense genotyping studies. Second, the missing gene expression data of the three associated genetic markers and their linked genetic regions rather than their own allele or genotype frequencies might indeed be the real etiological players in the pathogenesis of celiac disease. However, the biology of complex diseases is certainly much more complex than a direct SNP altered gene function disease relationship, and we must be very cautious when proposing celiac causal genes and pathogenic mechanisms based only on statistical association. Third, the sample size of this study is not large enough to draw a firm conclusion, such that our findings can be generalized to all Arab population. Thus to validate whether the GWAS findings are useful in identifying the celiac disease relevant etiological genetic markers, we recommend further studies involving larger population, extensive SNP coverage assays, and in vitro functional studies.

## 5. Conclusion

In conclusion, this study reports for the first time that MMEL1 518 Met/Thr polymorphism contributes to celiac disease risk among Saudi Arabians, both in single and also in synergistic cooperation with SH2B3 gene marker. Taken together, our study results have yielded mixed findings in replicating the European specific CD genetic susceptibility loci among a geographically and culturally distinct population from the Saudi Arabian Peninsula. In light of our results, this study further stresses the importance of identifying population-specific genetic susceptibility loci to obtain robust results that can be replicated. In particular, replication of GWAS loci association in populations like Saudi Arabians is relevant given their changing food habits with substantial dietary gluten component. To better understand the clinical implications of CD-specific GWAS loci, this study will further establish background data for further investigations into the mechanisms through which MMEL1 is likely to contribute in celiac disease development.

## Figures and Tables

**Table 1:** Characteristics of nonsynonymous SNPs screened in celiac patients.

Gene symbol	Gene name	RS ID	Alleles	Minor allele frequency	cDNA location	Genomic location	Exon	Protein effect
IRAK1	Interleukin-1 receptor-associated kinase 1	rs1059702	A/G	0.37	c.587 A > G	X:154018741	5	Phe196Ser

SH2B3	SH2B adaptor protein 3	rs3184504	T/C	0.22	c.784 T > C	12:111446804	2	Trp262Arg

MMEL1	Membrane metalloendopeptidase-like 1	rs3748816	T/C	0.46	c.1553 T > C	1:2595307	16	Met518Thr

**Table 2:** Genotype distributions and allele frequencies of examined polymorphism between patients and controls, as well as their prediction for celiac disease risk.

Gene	SNP ID	Genotype/allele	Cases (%)	Controls (%)	*x* ^2^	*P*	OR (95% CI)
IRAK1	rs1059702	AA	23 (23.71)	32 (25.8)	Reference		
		AG	24 (24.74)	38 (30.64)	0.1162	0.7321	0.87 (0.419–1.84)
		GG	50 (51.54)	54 (43.54)	0.5639	0.4539	1.28 (0.66–2.49)
		GG versus AG + AA	#	#	0.12	0.72	1.11 (0.60–2.07)
		AA versus AG + GG	#	#	1.3	0.23	0.72 (0.42–1.23)
		A	70 (36.08)	102 (41.12)	Reference		
		G	124 (63.91)	146 (58.87)	1.164	0.2815	1.238 (0.840–1.82)

SH2B3	rs3184504	TT	58 (59.79)	75 (60.48)	Reference		
		TC	36 (37.11)	42 (34.67)	0.077	0.78164	1.08 (0.618–1.895)
		CC	3 (3.09)	5 (4.83)	0.361	0.54813	0.64 (0.155–2.695)
		TT versus TC + CC	#	#	0.01	1	1.02 (0.59–1.77)
		CC versus TC + TT	#	#	0.42	0.51	1.59 (0.38–6.54)
		T	152 (78.3)	192 (78.68)	Reference		
		C	42 (21.7)	52 (21.31)	0.018	1	0.97 (0.616–1.527)

MMEL1	rs3748816	TT	38 (41.23)	55 (44.35)	Reference		
		TC	32 (32.98)	54 (43.54)	0.24	0.61	0.85 (0.47–1.56)
		CC^*∗*^	27 (25.77)	15 (12.09)	6.31	0.01	2.60 (1.22–5.54)
		CC versus TC + TT	#	#	0.59	0.43	1.23 (0.72–2.12)
		TT versus CC + TC	#	#	8.71^*∗*^	0.03^*∗*^	0.35 (0.17–0.71)
		T	108 (55.67)	164 (66.1)	Reference		
		C	86 (44.32)	84 (33.8)	5.01^*∗*^	0.02^*∗*^	1.55 (1.05–2.28)

^#^Genotype values are mentioned in above corresponding rows; ^*∗*^values are statistically significant.

**Table 3:** Summary of the interactive combinations of three examined polymorphisms by MDR analysis.

Genotype model	Cross-validation consistency	Testing accuracy	*P* value
MMEL1, c.1553 T > C	0.518	10	0.2061

MMEL1, c.1553 T > C + SH2B3, c.784 T > C	0.641	10	0.0156

IRAK1, c.587 A > G + SH2B3, c.784 T > C + MMEL1, c.1553 T > C	0.612	8	0.0786
